# Effects of Topically Applied Betulinic Acid and NVX-207 on Melanocytic Tumors in 18 Horses

**DOI:** 10.3390/ani11113250

**Published:** 2021-11-13

**Authors:** Lisa A. Weber, Julien Delarocque, Karsten Feige, Manfred Kietzmann, Jutta Kalbitz, Jessica Meißner, Reinhard Paschke, Jessika-M. V. Cavalleri

**Affiliations:** 1 Clinic for Horses, University of Veterinary Medicine Hannover, Foundation, Bünteweg 9, 30559 Hannover, Germany; lisa.annabel.weber@tiho-hannover.de (L.A.W.); Julien.delarocque@tiho-hannover.de (J.D.); Karsten.feige@tiho-hannover.de (K.F.); 2 Department of Pharmacology, Toxicology and Pharmacy, University of Veterinary Medicine Hannover, Foundation, Bünteweg 17, 30559 Hannover, Germany; Manfred.kietzmann@tiho-hannover.de; 3 Biosolutions Halle GmbH, Weinbergweg 22, 06120 Halle (Saale), Germany; kalbitz@biosolutions-halle.de; 4 Biozentrum, Martin-Luther-University Halle-Wittenberg, Weinbergweg 22, 06120 Halle (Saale), Germany; reinhard.paschke@biozentrum.uni-halle.de; 5 Equine Internal Medicine, University Equine Clinic, University of Veterinary Medicine Vienna, Veterinärplatz 1, 1210 Vienna, Austria

**Keywords:** equine melanocytic tumor, horse, oncology, skin neoplasia, topical drug, triterpenoids

## Abstract

**Simple Summary:**

Melanomas are skin tumors of the pigment-producing melanocytes. Equine melanomas are among the most frequently diagnosed tumors affecting grey horses. The melanocytic tumors progress to malignancy in more than two-thirds of cases. Previous laboratory experiments and studies with horses utilizing the naturally occurring betulinic acid (BA) and its derivative NVX-207 showed promising results with respect to the topical (epicutaneous) treatment of equine melanoma. Therefore, the aim of this feasibility study was to gain first insights into the effect and safety of BA and NVX-207 in eighteen horses with early-stage melanocytic tumors after a 13-week-long topical application. The topical treatment was convenient and safe. Compared to a placebo, the data suggest a positive treatment effect from topical application of BA and NVX-207 on equine melanomas toward the end of the treatment period. However, the time period studied was too short to conclusively prove this. Further advancement of the investigational medicinal products studied herein could lead to an effective, topical and marketable novel drug which helps to relieve suffering and, consequently, improve the welfare of equine skin cancer patients.

**Abstract:**

The naturally occurring betulinic acid (BA) and its derivative NVX-207 induce apoptosis in equine melanoma cells in vitro. After topical application, high concentrations of the substances can be reached in healthy equine skin. With the aim to investigate the effect and safety of topically applied BA and NVX-207 in horses with melanocytic tumors, the longitudinal, prospective, randomized, double-blind, placebo-controlled study protocol included eighteen Lipizzaner mares with early-stage cutaneous melanoma assigned to three groups. Melanocytic lesions were topically treated either with a placebo, 1% BA or 1% NVX-207 twice a day for 91 days. Caliper measurements, clinical examinations and blood tests were performed to assess the effects and safety of the medication. The topical treatment was convenient and safe. The volumes of tumors treated with BA were significantly reduced over time as compared to tumors treated with the placebo from day 80 of the study. Although treatment with NVX-207 seemed to decrease tumor volume, these results did not reach statistical significance. The findings must be regarded as preliminary due to the limited group size and need to be replicated in a larger cohort with modified pharmaceutical test formulations. Accordingly, the treatment protocol cannot yet be recommended in its current form.

## 1. Introduction

The susceptibility to melanoma development in grey horses is high due to genetic mutations [1,2]. Early stages of the melanomas located mainly in the dermis frequently occur as single, black-pigmented, firm nodules in glabrous skin under the tail root, around the anus, perineum, external genitalia, in the lips and eyelids, or in rare circumstances at other locations [3,4,5]. Economic and functional problems such as interference with harnessing and breeding impairment have been reported [6,7], but more severe and life-threatening visceral signs can occur with disease progression and metastasis [8,9,10,11,12,13]. The often slow-growing nature of the tumors, the proximity to important anatomical structures such as nerves, vessels or the anal sphincter, and the currently challenging or inefficient therapeutic options have led many practitioners to advocate benign neglect of small melanocytic tumor masses in horses [8,9]. However, every equine melanocytic neoplasm should be considered pre-cancerous or potentially malignant and, therefore, worthy of treatment [8,9,14]. The topical (epicutaneous) treatment of equine melanomas could be a feasible approach to treat early stages of the disease, aiming at preventing later malignant transformation. Topical therapies are characterized by their non-invasive nature and reduced systemic side effects [15,16]. Usually, they are affordable and can be performed with low logistical effort by the horse owners themselves, which reduces the stress factor on the horse significantly.

Betulinic acid (BA) is a pentacyclic lupane-type triterpenoid of plant origin [17]. Considerable amounts of the substance can be extracted from the bark of certain tree species, for example, the plane or the white-barked birch tree [17,18]. A wide range of pharmacological properties have been described for BA [19], among which the antitumoral features have been particularly studied [17,20,21]. The main antitumoral effects of the substance are based on the ability to trigger the mitochondrial pathway of apoptosis in cancer cells [22,23] to inhibit the eukaryotic topoisomerase I and II [24,25,26] and suppress the angiogenesis within the tumor [27,28,29,30]. Among a variety of BA derivatives, the compound NVX-207 has been identified as one of the most biologically active and pharmacologically significant agents [31,32,33]. The efficacy and mechanisms of BA and NVX-207 as potential therapeutics against equine melanoma were evaluated by in vitro cell culture experiments [33,34,35]. Reported findings suggest that BA and NVX-207 may achieve anticancer activity in equine melanoma cells due to cytotoxic and antiproliferative effects, whereby cell death is induced by apoptosis [33,34,35]. Concentration profiles of BA and NVX-207, both of which have been determined in vitro and in vivo, further indicated that the compounds’ half-maximal inhibitory concentrations for equine melanoma cells can be achieved in healthy horse skin [34,35,36]. The in vitro and in vivo studies reported provide a promising basis for the use of BA and NVX-207 as topical drugs in clinical trials for equine melanoma treatment [33,34,35,36]. Consequently, the aim of this longitudinal, prospective, randomized, double-blind, placebo-controlled pilot study was to gain first insights into the effect and safety of BA and NVX-207 in horses with early-stage melanocytic tumors after a 13-week-long topical application.

## 2. Materials and Methods

### 2.1. Approval of the Animal Experiments

The longitudinal, prospective, randomized, double-blind, placebo-controlled study protocol was approved by the institutional ethics and animal welfare committee of the University of Veterinary Medicine Vienna, Vienna, Austria and the Austrian Federal Ministry of Education, Science and Research in accordance with the Austrian Animal Welfare Law (BMBWF-Reference number: 68.205/0197-V/3b/2019). Informed consent was obtained from the stud management.

### 2.2. Horses

The study was performed between January and April 2020 at a stud farm in Austria. Eighteen white, flea-bitten or dappled Lipizzaner mares with cutaneous melanomas were included in this study (Table 1). The number of animals was determined by a power analysis using G*Power 3 [37] with the following assumptions: effect size 0.25, type I error 0.05, type II error 0.2 and correlation between measurements 0.75. Considering the planned duration of the trial, the relatively low division rate of the melanoma cells, the pharmacokinetic properties of the drug formulations and the relatively short intervals between measurements, the expected effect size was in the low to medium range and the expected correlation between measurements high. These parameters resulted in a total number of 15 animals. The estimated number of 18 animals included one additional animal per group, as at this low group size the exclusion of one animal would dramatically affect the power of the study. To avoid seasonal changes in the coat and skin (e.g., length and texture of the coat, possible sweating, sun exposure, wetness, exercise, etc.), the additional animals participated in the study from the beginning.

The median age of the horses was 14.5 years (range 6 to 28 years) and the median body condition score was 6 (range 4 to 8) according to the scoring scheme of Kienzle and Schramme [38]. None of the horses had ever been treated for melanoma in the past. Nine of the eighteen horses (horses 1, 5, 6, 9, 12, 13, 14, 15, 16) were in foal and the births of the foals were expected during or shortly after the study period. Horses were considered eligible for the study if they had cutaneous melanomas in localizations easy to treat (e.g., undersurface of the tail). Irrespective of the total number of melanomas identified on an individual horse, a maximum of two tumors per horse with a diameter of maximal 15 mm were treated. The tumors to be treated had to be easily distinguishable from each other and from other tumors. Clinical diagnosis was set at the beginning of the study on the basis of the localization and gross appearance of the lesions in conjunction with the horses’ signalment. Fine needle aspirations of tumor masses were performed in seven horses (horses 1, 5, 7, 12, 13, 17, 18) and revealed in 7/7 heavily pigmented cells, where the evaluation of nuclear criteria of malignancy was impossible. However, tumors were identified as pigmented lesions with unknown malignant potential. For the other horses, the procedure would only have been possible under sedation, which was not permitted by the stud management. Medical histories were obtained before the instigation of the topical treatment and a thorough physical examination was performed on each horse to ensure eligibility for the trial. The animals were kept in groups of 15 to 25 horses in stables overnight and on a paddock during the day. After birth, mothers and foals were separated in individual boxes for about seven days before they were kept together in groups with other mares and foals in large stables. All horses were fed a mix of muesli, oats and mineral feed daily, the quantity of which depended on body weight and performance. They had ad libitum access to hay and water.

### 2.3. Topical Treatment

**Figure 1:**
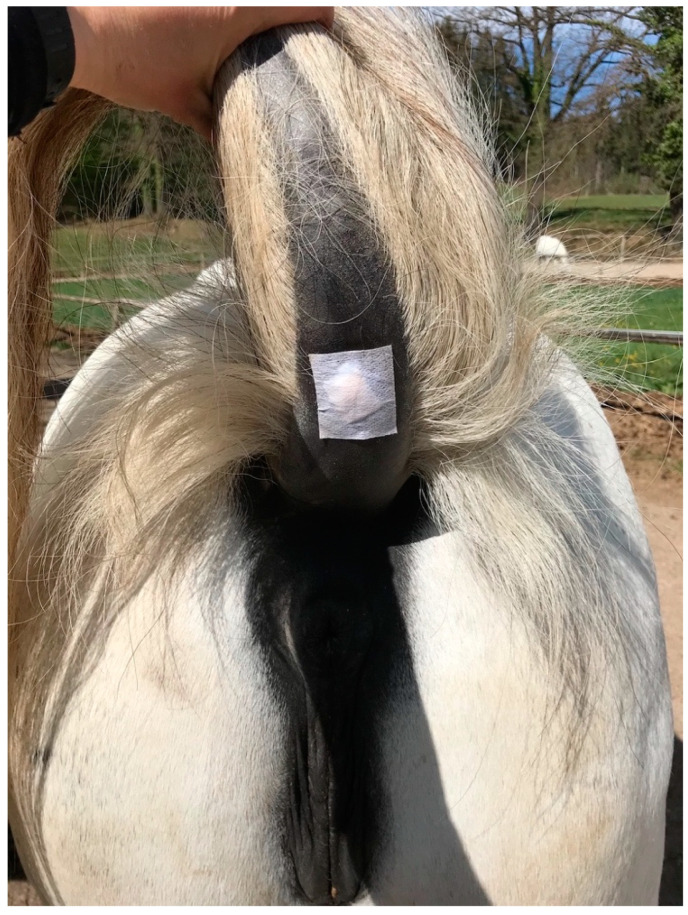
Covering the treatment site. Each tumor was treated topically with a placebo, a 1% BA cream or a 1% NVX-207 cream and subsequently covered in order to prevent the cream from being rubbed off. Any cream residues from previous treatments were removed with a swab once a day. If necessary, the skin was degreased with swabs soaked in 70% ethanol to ensure the fixation of the patches.

Melanomas were topically treated with pharmaceutical test formulations (creams) which had been previously tested for tolerability on eight healthy horses and in which a homogenous and stable distribution of BA and NVX-207 had been shown [36]. Treatment was performed twice daily and consisted of topical application of either 1% BA in “Basiscreme DAC” (amphiphilic cream as published in the German Drug Codex) with 20% medium-chained triglycerides, 1% NVX-207 in “Basiscreme DAC” or a placebo (“Basiscreme DAC” with 20% medium-chained triglycerides) for 13 consecutive weeks (91 days). Each tumor was completely covered with the cream and protected with an appropriately sized wound dressing (“Animal Soft” Snögg, Vennesla, Norway), which was fixed with “Fixomull stretch” (BSN medical GmbH, Hamburg, Germany) to prevent the cream from being rubbed off (Figure 1). As there are no safety studies on the topical application of BA and NVX-207 in humans, the person applying the cream wore gloves.

The study protocol stipulated that if the melanoma(s) disappeared completely before the end of the 13 weeks (tumor no longer palpable), the tumor was treated with the preparation assigned to it for another 14 days after tumor regression. The treatment was discontinued before the end of the 13 weeks if the tumor(s) grew aggressively.

The treatment was performed blinded by the first author (LAW). Horses 17 and 18 were treated by the stud’s staff from day 56 to 83 of treatment due to restrictions in the context of the SARS-CoV-2 crisis. The identical-appearing study medication was packed in identically number-coded jars. The patients were randomized into three groups (placebo, BA, NVX-207) of six horses. Randomization was performed by a person not involved in the study, assigning the horse numbers, which were written on paper sheets and blinded to him, to the blinded number-coded jars by random allocation. The numerical codes of the study medication were unblinded after all analyses had been completed.

### 2.4. Clinical Safety Assessment of the Treatment

The safety and tolerability of the topical treatment were evaluated by general clinical examinations, monitoring of the tumor and its surrounding clinically normal skin, assessment of the horses’ behavior during cream application according to [36], and hematologic and blood biochemistry profiles. The animals were examined clinically prior to each topical application (twice a day) in the first week of treatment. Thereafter, a general clinical examination was performed twice at 14-day intervals (day 21 and 35) and then twice at 30-day intervals (day 63 and 92). The melanoma to be treated and the surrounding tissue were assessed daily for local inflammation, ulceration and depigmentation. If observed, inflammatory skin reactions on treatment sites (redness, heat, swelling, pain) were classified into “mild”, “moderate”, and “severe” based on clinical experience. Blood was collected on days 0, 7, 21, 35, 63 and 92 for a complete blood count and serum chemistry profile, including electrolytes (sodium, potassium, chloride, calcium, magnesium), urea, creatinine, total protein, albumin, lactate, serum amyloid A and enzymatic activity of the alkaline phosphatase, glutamate dehydrogenase, g-glutamyl transferase and creatine kinase.

The study protocol specified that treatment was discontinued if the horse showed moderate skin changes in the area treated for more than two days, if the horse showed mild abnormal physical examination parameters for five days or moderate abnormal physical examination parameters for three days, and in the case of significant illness or general deterioration in the condition of the horse.

### 2.5. Tumor Response Evaluation

Target lesions were photographed and the length (mm, longest diameter) and width (mm, perpendicular to length) were measured with calipers (CONNEX GmbH, Oldenburg, Germany) prior to treatment (day 0) and on days 7, 21, 35, 63, 77 and 92. Measurements were performed in duplicate. Tumor volume (mm^3^) was calculated according to a formula described previously [39,40]: Tumor volume = length × width^2^ × 0.5. All tumors were measured by the first author (LAW). Follow-up examinations of the horses and tumors were performed four months after the last treatment.

### 2.6. Statistical Analysis

A generalized additive model of the Gamma family with log link function was fitted using R 4.0.2 [41] and the ‘mgcv’ package [42] to estimate trends in tumor growth during the experiment. The model consisted of a smooth function of time interacting with group (treatment), which was also included as a main parametric effect. The individual melanomas were considered random effects nested within horse. The inclusion of a separate random effect for ‘horse’ was discarded based on the higher Akaike information criterion (AIC) of the corresponding model. The thin plate regression splines were parametrized by restricted maximum likelihood [43]. The fitted smooth functions were compared on the link scale for each pair of groups via the use of a prediction matrix, as described in [44]. The given confidence intervals correspond to approximate 95% pointwise confidence intervals. P-values for parametric effects were obtained from Wald tests using the Bayesian covariance matrix for the coefficients. Normality of the residuals was ensured visually using histograms and quantile–quantile plots.

## 3. Results

### 3.1. Tumor Response

A total of 29 melanoma lesions (groups placebo n = 8; BA n = 12; NVX-207 n = 9) were treated twice a day for 13 weeks. Tumor diameters and volumes are described as medians and quartiles (QR; first quartile, third quartile). The median longest tumor diameter (length) measured on day 0 was 6.0 mm (QR; 5.0 mm, 7.5 mm) in the placebo group. Tumors in the BA group had a median length of 5.0 mm (QR; 5.0 mm, 8.0 mm). Tumor diameters in the NVX-207 group had QR of 5.0 and 6.0 mm with a median length of 5.0 mm. Supplemental data provide information on tumor length and width at the different measurement time points in the placebo group (Figure S1), BA group (Figure S2), and NVX-207 group (Figure S3).

**Figure 2:**
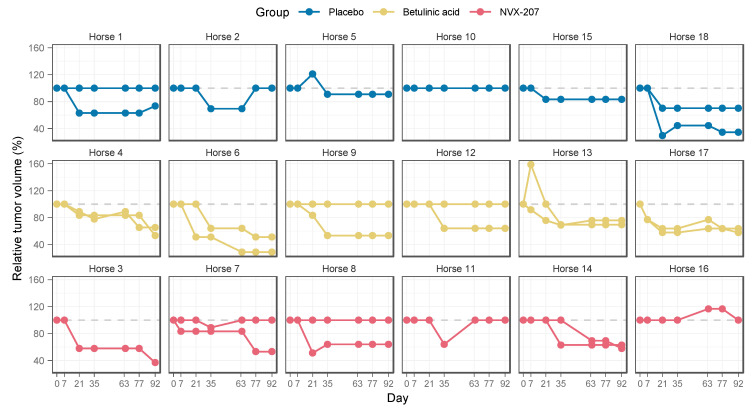
Relative tumor volumes over time for the individual horses. Equine melanomas were treated topically with a placebo, a 1% BA cream or a 1% NVX-207 cream twice daily for 91 consecutive days. The tumor volume before the initial treatment (day 0) corresponds to 100%, subsequent relative volumes are documented in percent of the baseline value. Depending on the number of tumors treated, one or two curves per horse are shown. In deviation from the statistical analysis, which is based on the absolute values, the values here are related to the baseline volume in order to achieve better visual comparability.

The median absolute tumor volumes in the placebo group reduced from 139.8 mm^3^ (QR; 68.8 mm^3^, 364.5 mm^3^) on day 0 to 117.0 mm^3^ (QR; 62.5 mm^3^, 191.0 mm^3^) on day 92. After treatment with BA, median tumor volumes decreased from 75 mm^3^ (QR; 62.5 mm^3^, 473.3 mm^3^) on day 0 to 51.3 mm3 (QR; 36.0 mm^3^, 322 mm^3^) on day 92. Median tumor volumes in the NVX-207 group decreased from 108.0 mm^3^ (QR; 62.5 mm^3^, 108 mm^3^) to 62.5 mm^3^ (QR; 40 mm^3^, 108 mm^3^). Figure 2 shows the relative tumor volumes over time for the individual horses.

**Figure 3:**
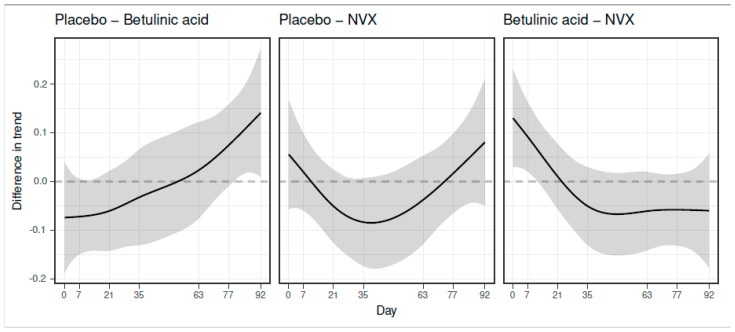
Pairwise differences between the smoothed curves of the different groups. Differences were formed from the predicted mean curves of tumor volumes and confidence intervals to visualize the time:group interaction. The difference between the groups named on top of the plots is significant when the dotted zero line lies outside of the confidence interval (in gray). For example, in the first half of the study period, the mean tumor volume in the BA group was larger than the mean tumor volume in the placebo group (resulting in negative values when the difference was formed). Thereafter, the volumes of tumors treated with BA were smaller than the volumes of tumors in the placebo group (resulting in positive values when the difference was formed). This difference was significant towards the end of the treatment period (zero line is outside the confidence interval from day 80). The difference between smooths is on the link scale and thus does not correspond to a difference in absolute tumor volume.

Disregarding time, no overall differences between groups were evident from the generalized additive model (Wald test: df = 2, F = 0.624, *p* = 0.537). In contrast, the group:time interaction distinguishes the time course of each group and may uncover differences at certain time points that are not sizeable enough to lead to an overall difference, but are still statistically significant and relevant to the interpretation. The group:time interaction indicated a significant difference in tumor volumes between groups BA and placebo from treatment day 80 (Figure 3). Although treatment with NVX-207 seemed to reduce tumor volumes in four horses (Figure 2), these effects did not reach statistical significance within the time:group interaction when compared to the placebo group (Figure 3).

No new melanocytic lesions were detected in any of the horses during the treatment period.

### 3.2. Clinical Safety Assessment of the Treatment

All horses tolerated the topical drug application well and no active defense movements were observed during the treatments. The dressings covering the treatment areas reliably remained at the desired location. Based on the clinical examinations of the horses, the topical melanoma treatment was safe in all groups. Two horses developed a mild spasmodic colic on day 7 (horse 7; NVX-207 group) and on day 13 (horse 17; BA group). Both cases of colic were successfully treated with a single administration of mild spasmoanalgesics (50 mg/kg bodyweight metamizole sodium IV plus 0.2 mg/kg bodyweight butylscopolammonium bromide IV; “Novasul” Richter Pharma AG, Wels, Austria and “Buscopan compositum” Boehringer Ingelheim, Ingelheim, Germany). All pregnant mares gave birth to healthy foals. The blood results revealed no hematologic toxicity or clinically relevant abnormalities at any time point.

**Figure 4:**
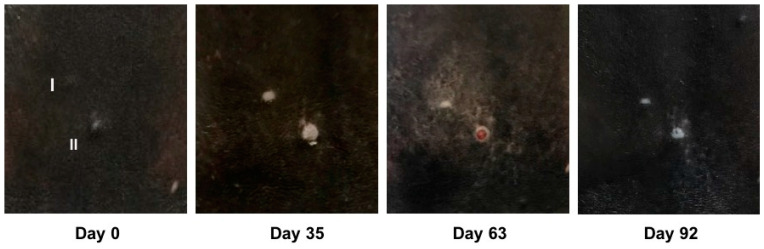
Clinical changes in melanoma I and II in horse 6 over time. The clinical changes in melanoma I and melanoma II (as indicated) on days 0, 35, 63 and 92 of the study. The tumors were treated twice a day with the 1% BA preparation (in “Basiscreme DAC” + 20% medium-chain triglycerides). In addition to the depigmentation of both tumors, an ulceration of melanoma II was occasionally observed from day 43 to day 70. Tumor volumes decreased from 63 mm^3^ (melanoma I) and 63 mm^3^ (melanoma II) on day 0 to 18 mm^3^ (melanoma I) and 32 mm^3^ (melanoma II) on day 92.

**Figure 5:**
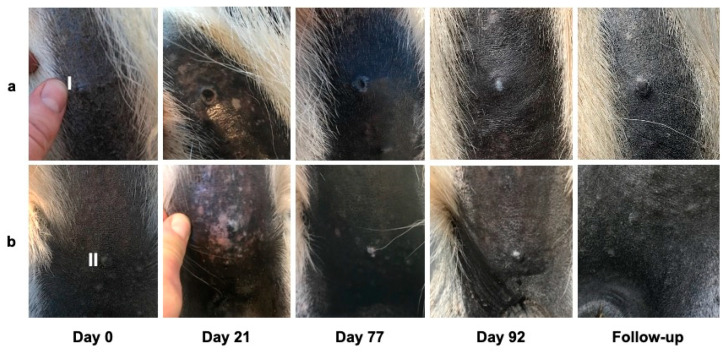
Clinical changes in melanoma I and II in horse 18 over time. The clinical changes in melanoma I (**a**) and melanoma II (**b**) on days 0, 21, 77 and 92 of the study and at follow-up examination. The tumors were treated twice a day with the placebo preparation (“Basiscreme DAC” + 20% medium-chain triglycerides). A crust formed on melanoma I on the 13th day of treatment. When the crust was removed on day 19, the skin was ulcerated and the tissue underneath the crust was black and surrounded by an epithelial border. The tumor had decreased noticeably in size. The area was completely covered with partially depigmented skin on day 84. There was a reduction in the tumor volume by 239 mm^3^ (melanoma I) and 109 mm^3^ (melanoma II) at day 92 compared to the baseline volume. An isolated to extensive depigmentation of melanoma II and the surrounding skin was observed from day 16. Depigmentation was a temporary side effect.

Depigmentation of the melanomas or the melanoma overlaying skin was observed in four out of eight tumors treated with the placebo (tumors of horses 1, 15, 18). The same was noted in 7 out of 12 tumors treated with BA (tumors of horses 4, 6, 13, 17) and three out of nine tumors treated with NVX-207 (tumors of horses 7 and 14). An ulceration of melanoma II was observed in horse 6 from day 43 to day 70 after treatment with BA (Figure 4). In horse 2, the melanoma-surrounding skin revealed isolated, depigmented areas from day 24 to day 86 of treatment. In horse 18, an isolated to extensive depigmentation and mild redness of the skin around melanoma II was observed from day 16 to day 30 (Figure 5). Apart from this, none of the horses showed clinical signs of redness, heat or swelling in the area of the treated skin.

Four months after the end of the last treatment, small, depigmented areas were apparent only in the two tumors of horse 17 (BA group). The skin of all the other horses was pigmented again.

## 4. Discussion

In the present pilot study, the topical application of 1% BA or 1% NVX-207 twice a day for 13 consecutive weeks in equine melanoma patients proved to be safe and was well tolerated. The topical therapy resulted in part in clinically visible and measurable changes in small melanocytic lesions, which were reflected in skin depigmentation and reduction in tumor diameters and volumes. A significant beneficial treatment effect could be shown after treatment with BA towards the end of the treatment period.

Although most melanocytic tumors in horses show a slow growth pattern for many years, more than two-thirds are thought to progress to malignancy [8,45]. For this reason, and because smaller tumors are easier to treat than large ones, even early (pre-cancerous) stages of equine melanoma should be subjected to therapy. Previously reported in vitro cell culture experiments and in vitro and in vivo permeation studies on unaltered horse skin indicated that the naturally occurring BA and its derivative NVX-207 may exert anticancer effects against equine melanoma [33,34,35,36]. The findings of this preceding work prompted further evaluation of the safety and efficacy of the compounds in equine melanoma patients in the current study. Smaller tumors were deliberately treated to explore a potential therapy that can be used for early stages of the disease. In these tumors, a cytological assessment of the cell nuclei for the criterion of malignancy could not be performed with certainty, as a consequence of the strong pigmentation of the cells. However, due to the small size of the melanocytic tumors, it can be assumed that they were pre-cancerous rather than malignant. It has been demonstrated previously that the calculation of tumor volumes with caliper measurement and the formula used here correlates well with tumor volumes calculated using three-dimensional ultrasound measurements [39,40]. Thus, caliper measurements were considered sufficiently reliable for tumor volume assessment.

A steady volume reduction was observed in most melanomas treated with BA. Despite the small number of cases, this resulted in a significant positive treatment effect in the time:group interaction compared to the placebo group towards the end of the treatment period. Although these first results after topical BA application on small equine melanocytic lesions are promising, the observations must be confirmed in larger studies with a longer treatment period and with a more diverse horse population in order to be able to draw sound conclusions regarding the effectiveness of the substance in melanoma-affected horses. Modifications in the test formulation, such as increasing the concentration of the active ingredient or incorporating permeation enhancers that transport large amounts of the compound through the fibrous tumor capsule of equine melanomas to the tumor cells, could also have a positive effect on tumor volume regression and may reduce the treatment period.

A cautiously positive, albeit not significant trend in tumor volume reduction was shown after 91 days of topical treatment with a cream containing 1% NVX-207. The treatment period investigated was too short, however, to prove this conclusively. Regarding the existing in vitro and in vivo data of NVX-207, it seems surprising that this derivative appears to have fewer anticancer effects on the tumors than its parent BA [31,33,34,35,36]. The substance’s reported in vitro half maximal inhibitory concentrations which lead to antiproliferative and cytotoxic effects in equine melanoma cells are much lower than those determined for BA [33,34,35]. However, it should not be disregarded that tumor cells integrated in their native microenvironment can be much more robust against pharmacological influences than tumor cells cultivated under in vitro two-dimensional cell culture conditions and, therefore, a reliable transferability of in vitro to in vivo results is not always given [46,47]. In addition, permeation barriers, such as the firm tumor capsule often found around equine melanocytic tumors, which could hinder the active substance to diffuse into the tumor cell, are also missing in vitro [5,46,48]. While the half maximal inhibitory concentrations determined for equine melanoma cells were surpassed after topical application of 1% NVX-207 in the epidermis, superficial and deep dermis of healthy horse skin [35,36], a less potent permeation into melanoma-affected skin could, therefore, further explain the only moderate effects of the compound in this study. It is also likely that tumors were located in the deep dermis and the NVX-207 applied topically may not have reached the full depth of the tumor invasion. An analysis of the NVX-207 content in the study medication a few weeks after the study termination revealed that the NVX-207 concentration had decreased only negligibly and a correlation between the reduced active ingredient content and reduced effectiveness can, thus, be excluded (own laboratory controls; data not shown).

The topical treatment of early stages of equine melanoma with 1% BA and 1% NVX-207 resulted in part in tumor volume and tumor diameter reductions and may represent—after modification of the pharmaceutical test formulations—an alternative to the frequently practiced approach of benign neglect of small solitary masses. Nevertheless, the results for lesions belonging to the BA and NVX-207 groups should be interpreted against the background that a few tumors in the placebo group also showed a decrease in size. It was stated previously that no reports about spontaneous melanoma regressions in horses exist [14,39]. When the growth behavior of 59 untreated melanomas was investigated in 17 Lipizzaner stallions from the same Lipizzaner stud as the Lipizzaner mares used in the present study, the tumor volume increased by 0.14% per day over an observation period of 162 days, but a slight reduction in tumor volume was sporadically observed in some lesions [49]. A trend in melanoma growth was observed in the placebo group of another study over only 64 days [50]. When the same pharmaceutical formulations as those used in this study were topically applied twice a day for seven days on eight healthy horses, an activation of the immune system by means of a perivascularly accentuated, lymphohistiocytic inflammation with a few neutrophils was observed in the superficial dermis of both the cervical and ventral tail skin [36]. As these alterations were noted in all treatment groups, an association with ingredients in the carrier cream “Basiscreme DAC” but no causative effect of the compounds BA or NVX-207 was suggested [36]. In the present study, the repeated topical application of the formulations for 13 consecutive weeks and the covering of the treatment areas could have led to an increased blood supply to the tumor area with increased immune cell infiltration. The presence of tumor-infiltrating lymphocytes has been associated with a favorable prognosis for human melanoma [51,52]. However, as no histopathological examinations of the melanomas treated with appropriate staining for vascularization markers or immune cell typing were performed in the present study, it remains unknown whether immunological adjuvant effects were involved in the tumor volume reduction. Since the tumor measurements were carried out by only one person, measurement variations can almost be excluded.

As there are currently no topical treatment options for equine melanoma that rely on larger clinical evidence-based studies, the treatment regime in the current study could only be presumed. In equine sarcoids, treatment durations between three and 45 weeks are reported for the topical approach [53,54,55,56]. Previously determined in vitro data indicated that in vivo treatment regimens with short application intervals and long treatment durations could favorably influence the concentration and efficacy of BA and NVX-207 in the skin of equine melanoma patients [34,35]. In addition, the application interval of 13 weeks utilized in the recent study is similar to an 11-week topical application of frankincense oil to an Arabian mare with stage 3 equine melanoma, which resulted in a clear tumor volume reduction [57]. The data generated in this pilot study suggest that an even longer treatment interval with 1% BA or 1% NVX-207 may lead to more notable clinical effects. However, this has not been definitively proven and, consequently, the study protocol applied in the current study cannot yet be recommended for melanoma therapy in horses. A treatment twice a day over a period of 91 days was feasible within the frame of the current study. Generally, for reasons of practicability, faster treatment success and owner compliance shortening the treatment period by improving the pharmacologic formulations should be the aim of future studies.

The topical melanoma treatment was safe and well tolerated in all groups as assessed by regular clinical examinations and serial blood sampling. The inconspicuous behavior may be related to the fact that the treatment did not cause painful skin inflammations. All horses showed an undisturbed general condition during the entire course of the study, apart from two horses with acute and medically resolved mild colic. Both horses that developed colic had a history of occasionally developing slight spasmodic colic at this time of the year. It seems very unlikely that the occurrence of colic was related to the topical melanoma treatment.

Depigmentation was occasionally observed in the skin surrounding and overlaying the tumors. The decreased amounts of melanin in the epidermis might be caused by toxic effects on the melanocytes or disturbed melanization due to the treatment [58]. Observations from follow-up examinations four months after the last treatment revealed that the depigmentation was a temporary side effect. While toxicity data of BA or NVX-207 for normal equine melanocytes are missing, a cytotoxicity of BA for human melanocytes by induction of apoptosis was described to varying degrees [59,60,61]. As the cases of depigmentation were also observed in the placebo group, an association with the ingredients of the amphiphilic carrier vehicle “Basiscreme DAC” is likely. With regard to the evidence of a mild skin irritative potential reported here and previously [36] and with respect to the fact that few tumors in the placebo group reduced also in size, it is recommended to use another pharmaceutical formulation as a placebo and vehicle for BA and NVX-207 in future studies.

The topical drugs used in this study are relatively affordable because the basic active ingredient BA is found in plants and is relatively inexpensive to produce. Although it is advantageous that topical medications are commonly low in cost and can be easily applied by horse owners, generally the benefits of topical therapies appear to be limited to only the lesions treated and no systemic antitumor effects can be achieved. Melanomas of the lip seem unsuitable for a topical therapy because of the risk that the animal will lick off the cream or ointment and absorb it orally. By contrast, the treatment of melanomas located on the ventral tail and in the perianal region has been proven to be very feasible in this study. Future trials should evaluate the effect, safety and feasibility of the topical medication investigated when applied to melanomas located in different anatomical regions.

Particularly because there is no established gold-standard treatment for equine melanoma, comparative study protocols should be considered for prospective studies. The phytochemical therapy introduced here and other described treatment modalities including surgery [62,63], radiation [64,65], (electro)chemotherapy [66,67,68] or immunotherapy [39,50,69,70] could be investigated. Moreover, approaches combining the aforementioned therapies with BA or NVX-207 as an adjunctive topical treatment could be the subject of further research.

The limitations of this pilot study include the usage of a single horse breed and sex, which limits predictability for a larger, more diverse population. In addition, due to the small number of animals, it was not further investigated whether the state of pregnancy in nine mares as well as the possibly different melanoma growth potential in horses with grey, flea-bitten or white coats could have an influence on the tumor response. Furthermore, no tissue samples of the melanomas treated and surrounding skin were taken, as this was not accepted by the stud management. Prospective studies should include tumor and skin biopsies in order to evaluate local treatment effects histopathologically. Another limitation of the study could be that the amount of cream applied per cm^2^ of tumor surface was not standardized. However, the application of only slightly different quantities of cream should not have had any effect on the results. On the one hand, there were always cream residues from the previous treatment, so more cream was applied per treatment than the skin could absorb. On the other hand, previous in vitro and in vivo studies showed that the concentration profiles of BA and NVX-207 hardly differ after 30 min and 24 h of incubation, at least in healthy equine skin, and that there is a reservoir effect due to the accumulation of the two substances in the *stratum corneum* [34,35,36]. Again, biopsies could be helpful in future studies to measure compound concentrations in tumor tissues.

## 5. Conclusions

The results presented in this pilot study indicate that topical treatment of early-stage equine melanoma with 1% BA and 1% NVX-207 twice a day over a period of 13 weeks is feasible and safe. Especially after BA application, positive effects were observed toward the end of the treatment interval. This suggests that this approach might be a potential therapy for early-stage equine melanoma and, thus, reduce the health risks associated with the possible malignant degeneration of the tumors. However, these findings must be regarded as preliminary due to the limited group size and need to be replicated in a larger cohort. Further, the long treatment duration could lead to poor owner compliance. Accordingly, the study protocol investigated in the current study cannot yet be recommended for melanoma treatment in horses. Modifications of the pharmaceutical formulations may further improve the clinical outcome and reduce treatment periods.

## Figures and Tables

**Table 1:** Characteristics and group assignment of the 18 Lipizzaner mares.

Horse ID	Treatment	Age (Years)	Color	Melanoma Stage ^1^	Number of Melanomas in Total	Number of Melanomas Treated	Localization of the Melanomas Treated
1	Placebo	19	white	2	>10	2	ventral tail
2	Placebo	14	white	2	5	1	ventral tail
5	Placebo	19	white	2	2	1	between tail root and anus
10	Placebo	9	dappled	2	>10	1	between tail root and anus
15	Placebo	9	flea-bitten	2	9	1	ventral tail
18	Placebo	28	white	2	>10	2	ventral tail
4	BA	17	white	2	3	2	ventral tail
6	BA	11	white	2	5	2	ventral tail
9	BA	12	flea-bitten	2	3	2	ventral tail
12	BA	18	white	2	4	2	ventral tail
13	BA	15	flea-bitten	2	>10	2	ventral tail
17	BA	27	white	2	>10	2	ventral tail
3	NVX-207	24	flea-bitten	2	>10	2	ventral tail
7	NVX-207	20	grey	2	>10	2	ventral tail
8	NVX-207	12	flea-bitten	2	3	2	ventral tail
11	NVX-207	14	grey	2	2	1	ventral tail
14	NVX-207	6	grey	2	>10	2	ventral tail + between tail root and anus
16	NVX-207	9	grey	2	>10	1	ventral tail

^1^ Disease staging according to Moore et al. [9]. Other locations where untreated melanomas occurred were the lip (horse 1), anus (horses 1, 3, 7, 17, 18), vulva (horses 3, 17, 18) and croup (horse 18). At the beginning of the study, all melanomas were covered by intact skin, except for a single ulcerated melanoma in each of horses 17 and 18 on the ventral tail (both lesions not treated within the frame of the study). The clinical examinations, hematology and blood chemistry were not indicative of internal metastasis in any of the horses.

## Data Availability

The datasets analyzed during the current study are available from the corresponding author on reasonable request.

## Supplementary Materials

The following are available online at https://www.mdpi.com/article/10.3390/ani11113250/s1, Figure S1: Tumor measurement values for the placebo group, Figure S2: Tumor measurement values for the betulinic acid group, Figure S3: Tumor measurement values for the NVX-207 group.

## Abbreviations

BA = betulinic acid; QR = quartiles.
