# Supplementary figures and images for: Effects of Topically Applied Betulinic Acid and NVX-207 on Melanocytic Tumors in 18 Horses
Source: Animals (Basel). 2021 Nov 13;11(11):3250. doi: 10.3390/ani11113250 (PMC8614291; doi:10.3390/ani11113250)

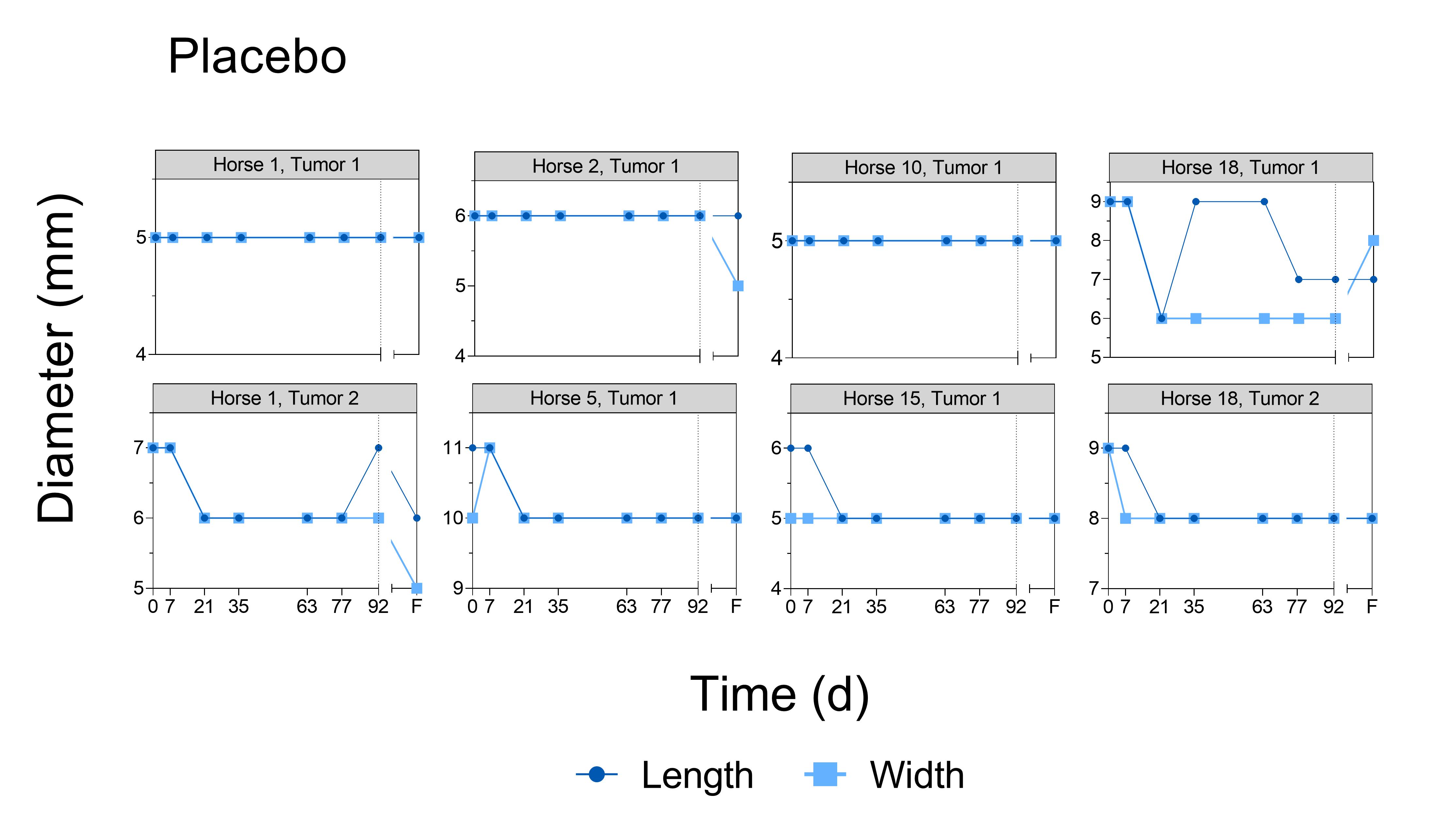

Supplement: Supplementary file 1 [file animals-11-03250-s001.zip › Figure S1_Supplemental data.jpg]

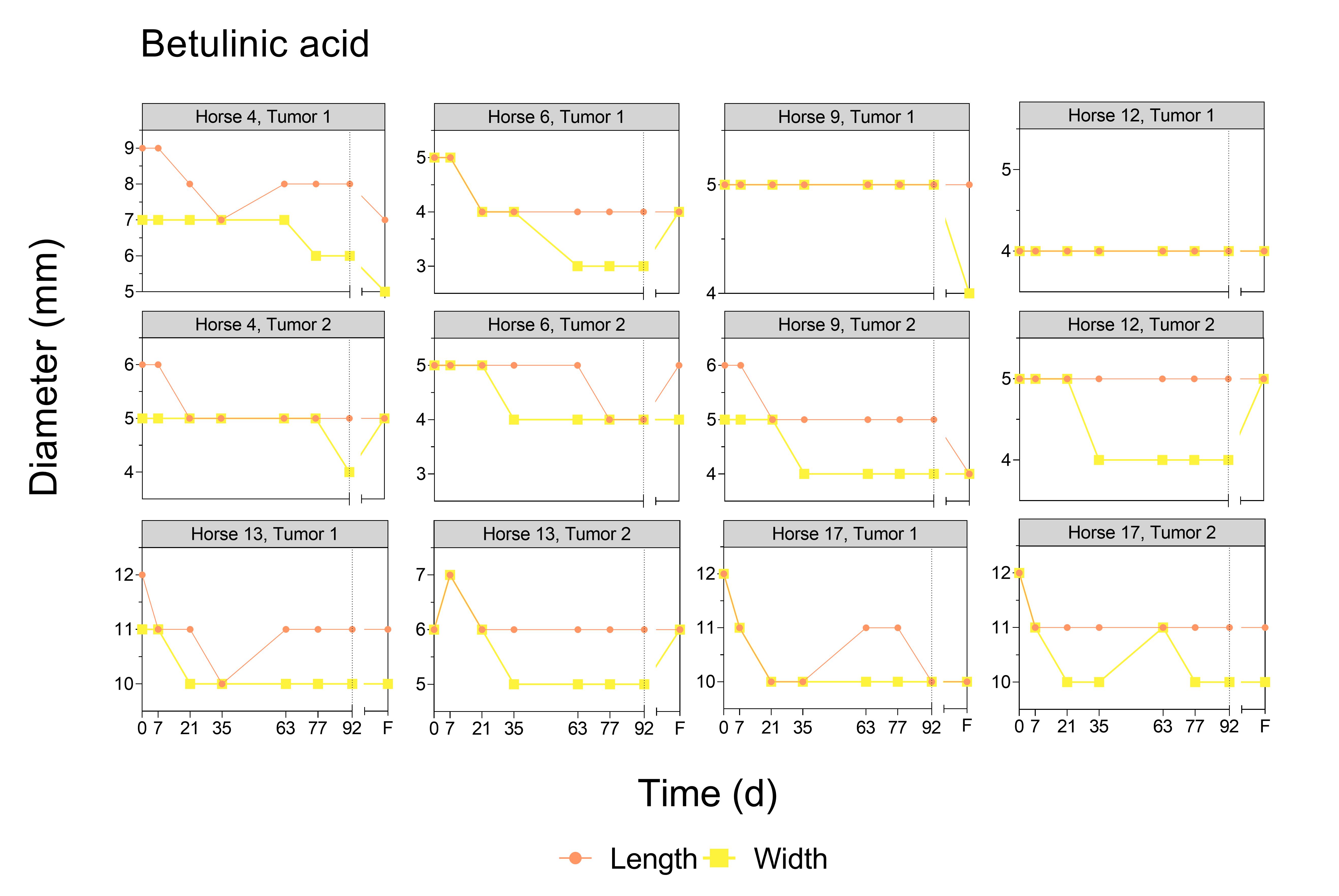

Supplement: Supplementary file 1 [file animals-11-03250-s001.zip › Figure S2_Supplemental data.jpg]

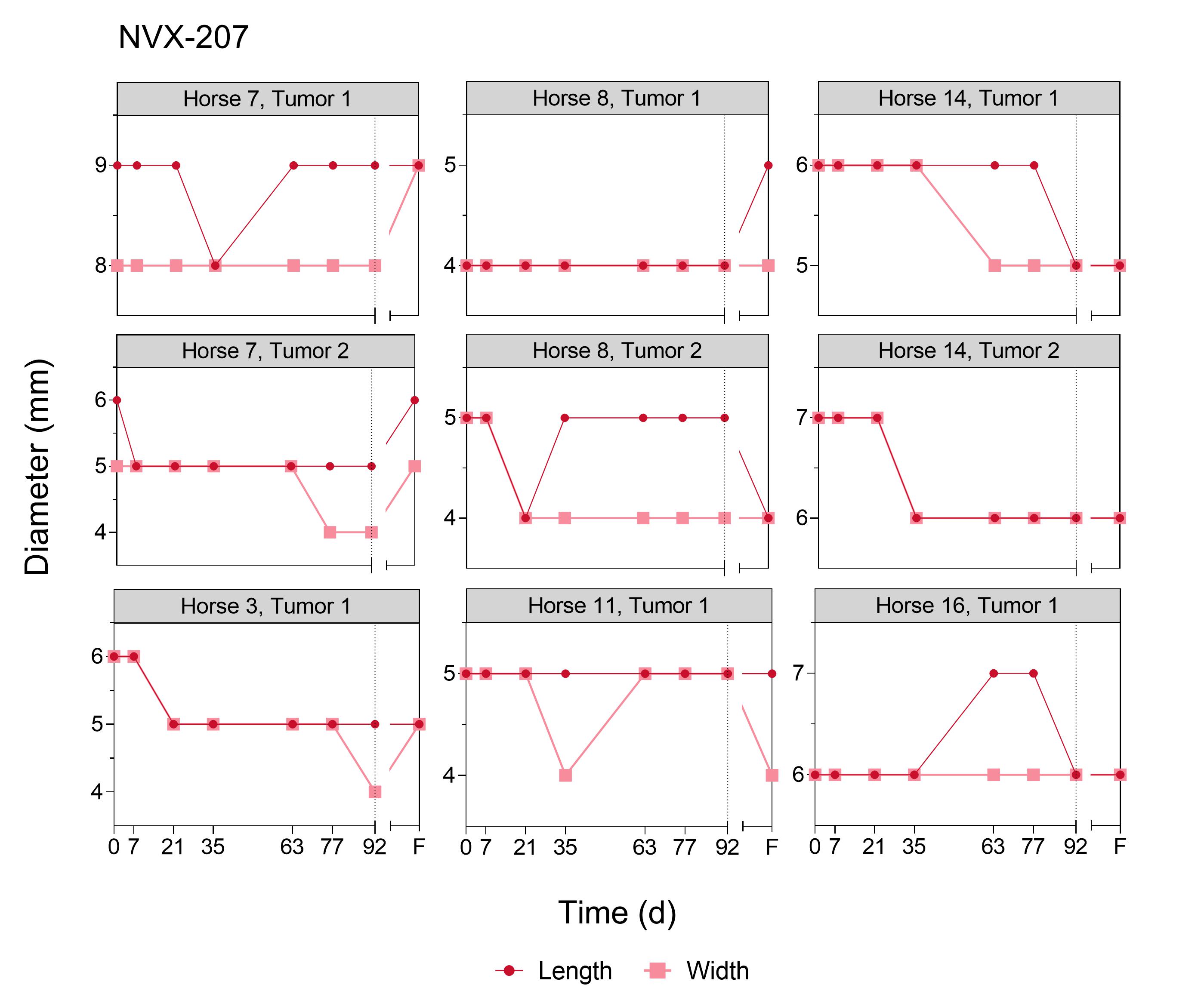

Supplement: Supplementary file 1 [file animals-11-03250-s001.zip › Figure S3_Supplemental data.jpg]
